# The influence of social alienation on maintenance hemodialysis patients’ coping styles: chain mediating effects of family resilience and caregiver burden

**DOI:** 10.3389/fpsyt.2023.1105334

**Published:** 2023-06-29

**Authors:** Qiaoling Liu, Li Zhang, Xia Xiang, Xiaoying Mao, Ying Lin, Jingfeng Li, Wen Cui

**Affiliations:** ^1^School of Nursing, College of Medicine, Shantou University, Shantou, China; ^2^Department of Office, First People’s Hospital of Foshan, Foshan, China; ^3^Department of Nursing, First People’s Hospital of Foshan, Foshan, China; ^4^Hemodialysis Center, First People‘s Hospital of Foshan, Foshan, China; ^5^College of Nursing, Zunyi Medical University, Zhuhai, China

**Keywords:** social alienation, family resilience, caregiver burden, coping style, maintenance hemodialysis

## Abstract

**Objective:**

Research on the possible impact of social alienation, family resilience, and caregiver burden on the coping styles of Chinese patients on maintenance hemodialysis (MHD) is scarce. We explore the influence of social alienation, family resilience, and caregiver burden on the coping styles of MHD patients, both directly and indirectly.

**Methods:**

We invited 173 MHD patients and their primary caregivers for a cross-sectional study; the study using convenience sampling method at the hemodialysis center of the First People’s Hospital of Foshan. The Chinese version of the generalized social of alienation scale, the Chinese version of the simplified coping style questionnaire, and a sociodemographic questionnaire were completed by the MHD patients, while their primary caregivers had filled out the Chinese family resilience assessment scale, the Chinese version of the Zarit caregiver burden interview, and provided socio-demographic information. SPSS macro program PROCESS v3.3 Model 6 were used for analyses of chain-mediated effects.

**Results:**

In the mediating effects model, the direct influence of social alienation upon coping styles was significant (95% CI −0.050, −0.014), and social alienation indirectly impacted coping style by family resilience in a significant way (95% CI −0.012, −0.001) or caregiver burden (95% CI −0.013, −0.001). In addition, social alienation significantly impacted coping style by both family resilience and caregiver burden (95% CI −0.008, −0.001).

**Conclusion:**

Social alienation can exert both a direct and indirect influence on coping styles through the mediating factors of family resilience and caregiver burden. Clinicians can take interventions to strengthen family resilience and reduce caregiver burden, which may be useful in improving socially isolated behaviors and coping skills in MHD patients.

## Introduction

Maintenance hemodialysis is essential to postpone disease progression and prolong the life of patients and is the most widely used alternative therapy for the treatment of end-stage renal disease (1). As reported by the most recent data (2), the prevalence of hemodialysis has been increasing over the past few years, and the current total prevalence of hemodialysis in mainland China was 402.18 per million. Maintenance hemodialysis is unable to fully compensate for a patient’s metabolic activity (3). MHD patients face many mental stresses during long-term treatment, such as heavy financial burden, negative body image and social isolation (4, 5). This series of negative impacts can cause individuals to withdraw, become alienated, or even develop social anxiety in interpersonal interactions. As a result, they may display social alienation behavior that prevents them from interacting positively with the outside world (6). Social alienation refers to a state in which individuals are unable to establish positive interactions with people or their surroundings, leading to negative emotions (7). Social alienation can be considered a stressor leading to mental stresses among chronic disease patients and is a predictor of various diseases and adverse health outcomes (8). However, this social alienation also increases the burden on their caregivers (9), families, and society (10). A recent study has shown that social alienation is an important factor leading to loneliness and depression (11), which can have an impact on the mental health and quality of life of the elderly (12). In addition, social alienation is one of the main risk factors for suicidal behavior (13). Therefore, it is important to explore how to maximize the return to life and social integration of MHD patients.

Coping is an attitude or action that individual takes in response to stress caused by changes in the internal and external environment (14). According to Lazarus’ stress response theory, individuals’ cognitive evaluations of stressors can vary, leading them to cope in either a positive or negative way (15). A recent study showed that coping styles are correlated with perceived stress, resilience, and social support (16). If a patient’s family possesses strong resilience or if caregivers provide more understanding and support, it can enhance the patient’s ability to cope with crises and adopt more proactive measures to alleviate negative stimuli. Positive coping is mainly characterized by the courage to confront difficulties and proactively seek solutions to problems. When patients have a positive coping style, it can assist them in better managing perceived threats and challenges, and give them a sense of control over their diseases (17), Additionally, regular engagement in social activities can foster positive psychological changes and a tendency towards positive coping styles in patients. Therefore, it is crucial to explore how social alienation affects coping styles in MHD patients.

Caregiver burden is described as the multifaceted stress perceived by caregivers as a result of providing care, and may further compromise the caregiver’s financial, physical, and mental health (18). The caregiver burden was associated with the patient’s conditions, including sociodemographic factors, mental status, disease progression, etc. (19). The caregiver burden was also related to daily caregiving hours, and the caregiver’s work status and sleep duration (20). At present, the burden of caring for MHD patients in China mainly rests on their family members. However long-term home care inevitably creates lasting stress on caregivers (21), and they may suffer from serious mental disorders and decreased care provision for patients (22). As a consequence, patients and their caregivers can hardly adapt to the new life status, and ultimately the whole family is involved, which leads to an imbalance in the family system (23, 24). In addition, a recent study showed that the caregiver burden is associated with negative psychological outcomes (25). Emotional issues faced by caregivers may cause patients to feel more isolated and helpless, leading them to adopt negative coping style. However, there have been no studies on the relationship between caregiver burden, social alienation, and coping styles.

When suffering hardships, some families failed to adapt well to changes and leading to deterioration in the quality of life, while some were able to cope well with adversity, one of the factors that determine whether the family adapts well was family resilience (26). Family resilience, as a family strength and power, is a protective factor for caregiver burden and has positive implications for promoting individual and family health. Family resilience helps caregivers to cope with the various challenges they face in long-term caregiving (27). However, family resilience was also vulnerable to multiple factors such as the patient’s disease progression (28), psychological status (29), disease perception (30), and caregiver burden level (31). Patients undergoing long-term hemodialysis treatment as a stressful event caused a huge impact on the whole family system. It was known through family resilience theory (32) that family resilience helps the whole family recover from distress and is critical for successful family adaptation to stressful events. Family resilience and caregiver burden may be able to influence patients’ social alienation and coping styles. However, the relationship between family resilience, social alienation, coping styles, and caregiver burden have not been confirmed.

Thus, we explore the chain mediating role of family resilience and caregiver burden between patients’ social alienation and coping styles at both individual and family levels. Based on the existing theory and literature, the research hypotheses are as follows: (H1) social alienation is negatively correlated with coping styles among MHD patients; (H2) family resilience has a mediating role in the relationship between social alienation and coping styles among MHD patients; (H3) caregiver burden has a mediating role in the relationship between social alienation and coping styles among MHD patients; (H4) family resilience and caregiver burden have a chain mediating role between social alienation and coping styles among MHD patients.

## Methods

### Participants

A total of 173 MHD patients and their primary caregivers at a hemodialysis center in a public hospital in China were invited to this study from September to October 2022. The inclusion criteria for the present study included: (1) patients should be at least 18 years old; (2) patients on regular dialysis for at least 3 months; (3) the caregiver must be a member of the patient’s immediate family; (4) participants have basic reading and expression skills and volunteered to participate in this study. The exclusion criteria included: (1) patients were diagnosed with mental disorders by physicians according to the Diagnostic and Statistical Manual of Mental Disorders (DSM-IV, TR) (33); (2) participants were unable to communicate or failed to complete questionnaires for some reasons; (3) patients combined with other serious life-threatening diseases, such as malignant tumors of other systems, cardiopulmonary failure, serious infections, etc.

### Procedure

After obtaining ethical approval from the First People’s Hospital of Foshan (No. 2022082), this study was performed in a hemodialysis center according to the 2013 revised Declaration of Helsinki. Before the survey began, participants were informed about the purpose of the study and the requirements to complete it and told that they could withdraw from the study at any time and only answer questions that they were comfortable with. All participants had signed written informed consent, which indicates that they are fully aware of the study procedures. Pen and paper self-report questionnaires were completed by patients and their primary caregivers in two separate quiet rooms before hemodialysis treatment. All self-report assessments were conducted by 2 trained assessors, and they were available to assist participants who had difficulty completing the questionnaire. These investigators were instructed to only read the items verbatim without providing any further explanation. The entire survey process lasted 15–20 min. After each questionnaire was completed, the evaluator reviewed the questionnaire immediately and demanded participants complete any missing items if they were comfortable with. A small gift was provided to all participants at the end of the survey, to compensate them for the time they took to complete the questionnaire.

### Measures

#### Socio-demographic information

The patients’ socio-demographic information included age, marital status, residence, living situation, education level, occupational status, medical insurance, duration of hemodialysis, and social contacts. The primary caregivers’ socio-demographic information included type of primary caregiver, age, education level, marital status, occupational status, monthly household income *per capita*, and duration of care.

#### Family resilience

The 20-item Chinese version of the Family Resilience Assessment Scale was used to measure family resilience (34), which has been tested in Chinese families for its psychometric properties, with a Cronbach’s *α* of 0.94. It comprises four subscales: perseverance, harmony, openness, and supportiveness. Each item was answered on a five-point Likert scale from 1 (very non-compliant) to 5 (very compliant), for an overall score range of 20 to 100, with higher scores indicating higher levels of family resilience. The Cronbach’s *α* for the scale in this study was 0.944, and the Cronbach’s *α* for each subscale was from 0.787 for openness to 0.900 for harmony.

#### Social alienation

The generalized social of alienation scale (GSAS) was developed by Jessor and his colleagues (35) to assess individuals’ feelings of alienation and uncertainty about participation in activities. In this study, the 15-item GSAS was used to measure social alienation (36), which was validated to have high validity and reliability, with a Cronbach’s *α* of 0.77. It comprises four subscales: the sense of social alienation, the sense of self-alienation, meaninglessness, and powerlessness. The score was answered on a four-point Likert scale from 1 (strongly disagree) to 4 (strongly agree), and the total score range was 15 to 60, with higher total scores indicating higher social alienation. The Cronbach’s *α* for the 15-item GSAS was 0.805, and the Cronbach’s *α* for each subscale ranged from 0.614 for powerlessness to 0.772 for feelings of self-alienation in this study.

#### Coping style

The coping style scale was developed by Folkman and Lazarus (37) to assess the coping styles of individuals. The 20-item Chinese version of the simplified coping style questionnaire (SCSQ) was used to assess coping styles (38), which has been tested for suitability in Chinese populations, with a Cronbach’s *α* of 0.90. The 20-item scale contains 2 dimensions: positive coping, and negative coping. The score was based on a four-point Likert scale ranging from 0 (not taken) to 3 (often taken). The higher score on which dimension, the more participants tend to adopt which coping style. The Cronbach’s *α* for the SCSQ in this study was 0.944, the Cronbach’s *α* for positive coping was 0.847 and for negative coping was 0.730.

#### Caregiver burden

The Zarit caregiver burden interview was developed by Zarit and his colleagues (39) to assess the caregiver burden of providing home care. In this study, the 22-item Chinese version of the caregiver burden inventory (CZBI) was used to measure caregiver burden (40). The scale has been tested in a Chinese sample and has high validity and reliability, with a Cronbach’s *α* of 0.87. It consists of 2 subscales: personal burden (12 items), and responsibility burden (6 items), and the remaining 4 items are independently scored. Each item was scored on a five-point Likert scale from 0 (never) to 4 (nearly always), and the total score range was 0 to 88, the higher scores indicating a greater burden of care. In this study, Cronbach’s *α* for the 22-item CZBI was 0.943, the reliability of the personal burden dimension was 0.847 and for responsibility burden dimension was 0.730.

#### Statistical analysis

All statistical analyses for this research were performed by using IBM SPSS 23.0 and SPSS macro program PROCESS v3.3. Descriptive data were described using means, standard deviations (mean ± SD), or frequencies (percentages). The correlations between family resilience, social alienation, caregiver burden, and coping styles were explored using Pearson’s correlation analysis. The chain-mediated effects analysis was performed using the SPSS macro program PROCESS v3.3 Model 6, and the significance of the mediated model was tested using the bias-corrected percentile Bootstrap method (5,000 resamples, 95% CI). *p* < 0.05 (two-tailed) was set as the statistical significance level for this study.

## Results

### Descriptive statistics

A total of 173 MHD patients and their primary caregivers participated in this study, and the descriptive statistics for all variables are shown in Table 1. Among the 173 patients, most were married (79.2%), with primary school education or below (54.9%), retired/unemployed status (79.2%), living in town (93.1%), normally socialized (60.1%), and had medical insurance (97.1%). Regarding the duration of hemodialysis, 80 (46.2%) were on dialysis for more than 3 years. The patients’ primary caregivers were spouses (60.1%), children (32.4%), parents (6.4%), and siblings (1.2%), and most of the primary caregivers had more than 5 years of care (53.2%), full-time status (42.8%), and monthly household income ranges from 3,000 to 6,000 RMB (49.1%).

**Table 1:** Socio-demographic information on MHD patients and their primary caregivers (*N* = 173).

Variables	Patients (*n* = 173)	Caregivers (*n* = 173)
	*N* (%)	*N* (%)
Age (years)		
19 < 45	33 (19.1)	56 (32.4)
45 < 65	65 (37.6)	76 (43.9)
65 < 80	64 (37.0)	37 (21.4)
≥80	11 (6.4)	4 (2.3)
Educational level		
Primary school or below	95 (54.9)	68 (39.3)
Secondary school	54 (31.2)	55 (31.8)
University or above	24 (13.9)	50 (28.9)
Marital status		
Single	9 (5.2)	19 (11.0)
Married	137 (79.2)	142 (82.1)
Divorced/widowed	27 (15.6)	12 (6.9)
Occupational status		
Full-time job	29 (16.8)	74 (42.8)
Part-time job	7 (4.0)	17 (9.8)
Unemployed/retired	137 (79.2)	82 (47.4)
Residence		
City/suburban	113 (65.3)	
Countryside	60 (34.7)	
Living status		
With family	161 (93.1)	
Alone	12 (6.9)	
Medical insurance		
Yes	168 (97.1)	
No	5 (2.9)	
Duration of hemodialysis (months)		
<6	14 (8.1)	
6 < 12	24 (13.9)	
12 < 36	55 (31.8)	
≥36	80 (46.2)	
Social contact		
Normal	104 (60.1)	
Less	60 (34.7)	
Avoid	9 (5.2)	
Primary caregiver		
Spouse		104 (60.1)
Child		56 (32.4)
Parent		11 (6.4)
Sibling		2 (1.2)
Duration of care		
3 months < 1 year		27 (15.6)
1 < 3 years		31 (17.9)
3 < 5 years		23 (13.3)
≥5 years		92 (53.2)
Monthly household income *per capita* (RMB)		
<3,000		33 (19.1)
3,000 < 6,000		85 (49.1)
6,001 < 10,000		34 (19.7)
≥10,000		21 (12.1)

### Correlation between family resilience, social alienation, caregiver burden and coping style

The results of descriptive statistics and bivariate correlations for family resilience, caregiver burden, social alienation, and coping styles are shown in Table 2. A negative correlation was found between social alienation and family resilience (*r* = −0.292) and between social alienation and coping style (*r* = −0.371), while a positive correlation was found between social alienation and caregiver burden (*r* = 0.341), both at the 1% significance level. A negative correlation was found between family resilience and caregiver burden (*r* = −0.503), while a positive correlation was found between family resilience and coping style (*r* = 0.347), both at a 1% significance level. A negative correlation between caregiver burden and coping style (*r* = −0.379) was found at the 1% significance level.

**Table 2:** Correlation of social alienation, family resilience, coping styles, and caregiver burden in MHD patients (*N* = 173).

	Mean ± SD	Range	Family resilience	Social alienation	Coping style	Caregiver burden
Family resilience	77.49 ± 11.55	69 (29–98)	1.000			
Social alienation	35.03 ± 5.92	35 (16–51)	−0.292^**^	1.000		
Coping style	30.96 ± 8.92	51 (2–53)	0.347^**^	−0.371^**^	1.000	
Caregiver burden	23.53 ± 15.82	74 (2–76)	−0.503^**^	0.341^**^	−0.379^**^	1.000

^**^p < 0.01.

### Chain mediation effects analysis

**Figure 1:**
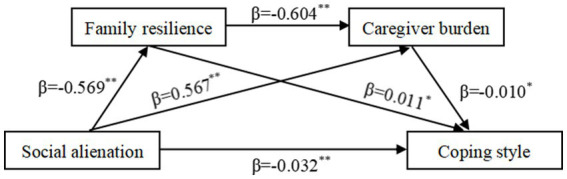
Chain mediating effects of family resilience and caregiver burden between social isolation and coping styles in MHD patients. **p < 0.01, *p < 0.05.

A chain mediation model was developed with social alienation as the independent variable, family resilience and caregiver burden as mediating variables, and coping style as the dependent variable. An analysis of the chain mediation model between the four variables were depicted in Figure 1. The results of the test for chain-mediated effects of family resilience and caregiver burden on social alienation and coping styles are presented, respectively, in Tables 3, 4. The results indicated that: (a) the direct influence of social alienation upon coping styles was significant, and the value of direct influence was −0.032 (95% CI −0.050, −0.014); (b) social alienation indirectly impacted coping style through family resilience in a significant way, the value of indirect influence was −0.006 (95% CI −0.013, −0.001); (c) social alienation indirectly impacted coping style through caregiver burden, and the value of indirect influence was −0.006 (95% CI −0.012, −0.001); (d) social alienation significantly impacted coping style through both family resilience and caregiver burden, the value of indirect influence was −0.003 (95% CI −0.008, −0.001). The bias-corrected 95% CI for all pathways did not contain 0, which was statistically significant.

**Table 3:** Regression analysis of variable relationships in chained mediation models (*N* = 173).

Variables	Family resilience			Caregiver burden			Coping style		
	*β*	SE	*t*	*β*	SE	*t*	*β*	SE	*t*
Social alienation	−0.569	0.143	−3.987^**^	0.567	0.180	3.153^**^	−0.032	0.009	−3.459^**^
Family resilience				−0.604	0.092	−6.554^**^	0.011	0.005	2.134^*^
Caregiver burden							0.010	0.004	−2.595^*^
*R* ^2^	0.085			0.295			0.231		
*F*	15.897^**^			35.487^**^			16.884^**^		

^**^p < 0.01, ^*^p < 0.05.

**Table 4:** Path analysis of mediating effect among four variables.

Pathways	Effect	SE	95% CI		Percentage of mediating effect %
			LLCI	ULCI	
Social alienation → coping style	−0.032	0.009	−0.050	−0.014	68.08
Social alienation → family resilience → coping style	−0.006	0.003	−0.013	−0.001	12.77
Social alienation → caregiver burden → coping style	−0.006	0.003	−0.012	−0.001	12.77
Social alienation → family resilience → caregiver burden → coping style	−0.003	0.002	−0.008	−0.001	6.38
Total mediating effect	−0.015	0.004	−0.024	−0.008	31.92
Total effect	−0.047	0.009	−0.065	−0.029	100

## Discussion

We explored the influential pathways of coping styles of MHD patients in terms of the family’s intrinsic factors and caregivers’ external influences. We tested the effects of family resilience and caregiver burden as chain-mediated variables on social alienation and coping styles. The results of the mediated effects analysis indicated that social alienation directly affected the coping style of MHD patients. Moreover, social alienation also indirectly affects the coping styles of MHD patients both through family resilience and caregiver burden. The indirect effects include three mediating pathways: (a) family resilience as a mediating variable; (b) caregiver burden as a mediating variable; (c) chain-mediated pathways with family resilience and caregiver burden as mediating variables.

In this study, social alienation in MHD patients was at a moderate level (35.03 ± 5.92), which is lower than the level (41.57 ± 4.89) (41) of maintenance hemodialysis patients in China. There are several reasons why MHD patients have a moderate level of social alienation. First, the preventive and control measures for the COVID-19 pandemic have been escalating, which reduced opportunities for social interaction and increased the social alienation of MHD patients. Second, two to three per week of maintenance hemodialysis, higher level of mobility, and treatment-related self-image disturbances (42) probably limit the ability of MHD patients to participate in social activities, in other words, this does not facilitate healthy interaction with the outside world. Lastly, it is important to acknowledge that MHD patients typically rely on the support and assistance of caregivers, including family members or friends. However, the caregiving role often places significant physical, psychological, financial, and time-related burdens on caregivers. These burdens can contribute to emotional fatigue and psychological stress (22), which may hinder caregivers from providing optimal support and care to patients. Consequently, the social alienation experienced by MHD patients may be further exacerbated.

Our results support the hypothesis that social alienation negatively correlates with the coping styles of MHD patients. MHD patients tend to adopt a negative coping style when social alienation levels increase. Social alienation is quantifiable for indicating the shrink of social networks and reduce of social connections (43). After staying away from social activities for a long time, patients lose the support of social groups, which in turn aggravates their withdrawal and avoidance and eventually could cause inability to communicate with others. Coping abilities are necessary for people to build healthy social networks. According to Roy et al. (44), human develop adaptive responses when facing stress, and the physiological and psychological regulation of human is mainly expressed through coping behaviors adaptively. A positive coping style can be helpful for patients to maintain normal social activities. Therefore, helping MHD patients to cope better with the struggle of chronic illness and maintain normal social interactions, which is significant for developing strategies to reduce the social alienation of MHD patients.

We validated the mediating effect of family resilience between social alienation and coping styles. Recent research indicated that family resilience was positively correlated with coping styles and had a direct predictive effect on patients’ self-care status (45). Family resilience can enhance the ability of family members to cope with adversities and has positive implications for solid family function (46). In this study, MHD patients with higher levels of family resilience were able to manage stressful events better, they developed positive coping styles and reduced social alienation levels while overcoming adversities. Therefore, we recommend that clinicians and nurses pay attention to assessing family resilience levels in MHD patients and developing family resilience-based interventions to promote their positive coping with the negative emotions that arise during long-term hemodialysis treatment.

Our results also validated the mediation effect of caregiver burden between social alienation and coping styles. Caregiver burden is a mediating variable, which mitigates the effect of social alienation on coping styles of MHD patients. In this study, reducing the caregiver burden helped patients build active coping styles for long-term hemodialysis treatment, which was consistent with previous results (47). At the same time, patients and caregivers are emotionally connected and negative emotions of them can lead to negative changes in family relationships (48), these negative changes partially lead to socially alienated behaviors and increase the burden on caregivers, which in turn affects the coping strategies of MHD patients. Therefore, it is necessary to focus on family counseling programs and dualistic interventions that combine patients and caregivers to deal with their negative emotions.

Finally, our results suggest that family resilience and caregiver burden play significant chain mediating roles among social alienation and coping styles. Previous research has illustrated that family resilience and patients’ emotional symptoms were influential factors in caregiver burden because these two factors impact the stress level of the caregiver (49, 50). When MHD patients have higher levels of family resilience, they can better coordinate family relationships, social resources, and support to cope with the adverse effects of long-term treatment. Higher levels of family resilience also promote positive coping and normal social interaction in MHD patients and reduce the burden on caregivers. However, it is worth noting that the indirect influence of family resilience and caregiver burden was relatively modest, suggesting the possibility of additional mediating variables between social alienation and coping styles. Future studies could delve into exploring these potential mediating variables, expanding our understanding of the complex relationship between social alienation and coping styles.

### Limitations

The limitations of this study are as follows. Firstly, this study is a cross-sectional study, thus, it cannot infer a causal relationship among social alienation, family resilience, coping styles, and caregiver burden. Secondly, convenience sampling may lead to selection bias, which limits the applicability of the conclusion. Therefore, a multicenter investigation is necessary for future studies. Thirdly, although we reviewed clinical records and asked patients about their mental health history at the beginning of the study, it was insufficient that we did not conduct a standardized interview to assess, the data we collected from the questionnaires may be biased by mental status. Finally, only caregivers reported family resilience in this study, which may not be comprehensive enough to characterize the resilience level of the whole family. Family resilience can be explored in the future from the perspective of all family members.

## Conclusion

On one hand, social alienation directly influenced coping styles, on the other hand, it also influenced indirectly the coping styles of MHD patients through family resilience and caregiver burden. In addition, family resilience and caregiver burden have significant chain mediating roles between social alienation and coping styles. Our findings emphasized the importance of three mediated paths between social alienation, family resilience, caregiver burden, and coping styles. Our findings also support the idea that assessing and enhancing family resilience, as well as reducing caregiver burden is critical to changing socially alienated behaviors and improving the coping skills of MHD patients. Therefore, clinical practitioners should pay attention to assessing the level of family resilience in MHD patients, and the family-based intervention combining patient and caregiver can be developed to deal with the negative emotions of long-term treatment and improve the quality of life for patients and their caregivers.

## Data availability statement

The original contributions presented in the study are included in the article/supplementary material, further inquiries can be directed to the corresponding author.

## Ethics statement

The studies involving human participants were reviewed and approved by the Ethics Committee of First People’s Hospital of Foshan (No. 2022082). The patients/participants provided their written informed consent to participate in this study.

## Author contributions

QL: data collection, methodology, writing original draft, writing review, and editing. XX, XM, YL, JL, and WC: data collection, data curation, and provison of study materials. LZ: conceptualization, project administration, writing review, and editing. All authors contributed to the article and approved the submitted version.

## Conflict of interest

The authors declare that the research was conducted in the absence of any commercial or financial relationships that could be construed as a potential conflict of interest.

## Publisher’s note

All claims expressed in this article are solely those of the authors and do not necessarily represent those of their affiliated organizations, or those of the publisher, the editors and the reviewers. Any product that may be evaluated in this article, or claim that may be made by its manufacturer, is not guaranteed or endorsed by the publisher.
